# Will you accept a virtual human as a friend? Exploring the role of virtual humans in mood management and mental health support

**DOI:** 10.3389/fpubh.2025.1555218

**Published:** 2025-04-04

**Authors:** Doyeon Lee, Byeng-Hee Chang, Sylvia Chan-Olmsted

**Affiliations:** ^1^Department of Psychology, University of Florida, Gainesville, FL, United States; ^2^Department of Media and Communication, Sungkyunkwan University, Seoul, Republic of Korea; ^3^Department of Media Production, Management, and Technology, University of Florida, Gainesville, FL, United States

**Keywords:** virtual human, mood management, cognitive behavioral theory of PIU, Technology Acceptance Model, Uncanny Valley Effect

## Abstract

**Introduction:**

The advancement of virtual human technology has opened new possibilities for its application in various fields, including healthcare. Recent research has begun exploring the use of virtual humans as tools for mood management in daily life. However, there is limited understanding of how individuals perceive virtual humans for this purpose, particularly in social media contexts where they are often viewed as influencers rather than therapeutic entities. This study investigates whether people intend to accept virtual humans as a kind of friend and engage in mood management. The study applies the Cognitive Behavioral Theory of Pathological Internet Use integrated with the Technology Acceptance Model and the Uncanny Valley Effect to explain why people who prefer online social interaction use virtual humans for mood management, and to examine whether they will accept virtual humans as friends.

**Methods:**

This study employs survey, where participants were asked about their perceptions of virtual humans, specifically focusing on their willingness to accept virtual humans as friends for mood management on social media platforms. The sample consists of social media users who are familiar with virtual influencers. A series of hypotheses were tested using structural equation modeling to assess the relationships between prefer for online social interaction, online chatting mood regulation, perceived usefulness, ease of use, and intention to accept virtual humans for mood management, as well as the moderating effect of perceived eeriness.

**Results:**

The study found that the preference for online social interaction has a positive impact on using online chatting for mood management, both for casual chatting and therapeutic chatting. Individuals seeking casual chatting perceive usefulness and ease of use for virtual humans, while those seeking therapeutic chatting perceive only usefulness. Perceived usefulness and ease of use have a positive effect on the intention to accept virtual humans as friends. However, perceived eeriness does not have a moderating effect between the variables.

**Discussion:**

The study highlights the potential of virtual humans to assist with mood management on social media platforms, indicating that they could serve a practical role in psychological well-being. While the current study suggests that virtual humans are seen as useful for therapeutic purposes, challenges remain in helping users envision them in this role. Future research should address these challenges by exploring the nuances of user interaction with virtual humans and examining their potential to make a broader social impact, especially in the fields of mental health care and personalized customer service.

## Introduction

With the development of technology, the creation of human-like virtual characters, also known as virtual humans, has advanced significantly (1). One of the strongest points of virtual humans is that they are not subject to time and physical constraints; they do not require management personnel, and they are not affected by external conditions such as weather or health (2). Therefore, it is evaluated as efficient to integrate them into various industries, as they are unlikely to be hindered by sudden external human factors. Consequently, virtual humans are being implemented in various sectors, with the healthcare field actively exploring their potential applications, including as virtual therapists and counselors (3–5).

In light of this usability, this study focuses on the necessity to increase efficiency and effectiveness by expanding the scope of application of virtual humans, making them more accessible not only in the professional medical healthcare field but also in daily life. Despite the potential for utilization, existing research on virtual humans in the healthcare field primarily focuses on professional medical areas (6–9). As an initial study with this purpose, this research will conduct an exploratory examination of whether people have the intention to accept virtual humans in daily life, assisting users in improving mental health, also known as wellbeing, through interaction. Specifically, this study aims to examine whether people are willing to accept virtual humans existing in social media as a type of friend and engage in mood management through interaction with these virtual humans.

To ensure relevance, this study focused on individuals who have a high potential to experience well-being using online virtual humans in daily life. It is judged that this group, which finds well-being through online virtual human interaction, consists of individuals who prefer online social interactions. Previous studies have shown that individuals who prefer online social interaction often engage in mood management through online chatting (10, 11). This study aims to examine whether individuals who prefer online social interaction have the intention to accept virtual humans as friends who can assist them with mood management, mediated by perceived usefulness and perceived ease of use. However, according to the Uncanny Valley Effect (UVE), the perceived eeriness of human-like objects can evoke unpleasant feelings (12), potentially influencing the decision to accept them as friends (13). Therefore, this study will also verify the moderating effect of perceived eeriness on the relationship between perceived usefulness, perceived ease of use, and the intention to accept virtual humans as friends who can assist in mood management.

The theoretical background of the influences between variables includes Problem Behavior Theory (PBT) (14), Technology Acceptance Model (TAM) (15), and the Uncanny Valley Effect (UVE) (16), which will be discussed in the literature review section. This study integrates the theories of PBT, TAM, and UVE to present a novel approach. First, PBT is a theory that explains negative behaviors such as internet addiction. However, in this study, we introduce a new perspective by utilizing TAM’s variables of perceived usefulness and ease of use to transform the internet addiction behavior described by PBT into a potential for effectively utilizing online technology. This plays a crucial role in the acceptance of virtual humans. Additionally, given that the subject of this study is virtual humans, we incorporate UVE, which explains the phenomenon of unpleasant feelings when virtual humans resemble humans, and explore how the cognitive and technological factors examined through PBT and TAM are influenced by the appearance of virtual humans.

## Literature review and hypotheses

### Problem Behavior Theory

The Problem Behavior Theory (PBT), proposed by Jessor and Jessor (14), helps to explain the nature of dysfunction and maladaptive behaviors. According to the theory, problem behavior can be defined as risky behaviors that interfere with psychological wellness, and these behaviors are considered to pose threats to health and well-being (17). Therefore, studies on problem behaviors, including alcohol addiction, eating disorders, smoking, drug use, and gambling, have applied PBT to explain these behaviors (18–20). With the widespread use of media devices due to the development of IT technology, internet access has become more convenient, and internet addiction is also considered a problem behavior that can negatively affect psychological wellness. There have been attempts to understand internet addiction behavior using PBT (21). This study focuses on internet addiction behavior based on PBT. However, this study acknowledges that while dependence on the internet may have negative impacts, there are also positive aspects in being able to effectively utilize new media technologies. It emphasizes the characteristics of internet addiction behavior and seeks to explain the intention of internet-dependent individuals who prefer online social interaction over offline person-to-person interaction to accept virtual humans as friends for mood management.

To understand individuals who are internet-dependent and prefer online social interaction, the concept of Pathological Internet Use (PIU) needs to be explained. PIU, proposed by Davis (22), was introduced to elucidate the mechanism by which internet-dependent problem behavior occurs. According to Davis (22), PIU can be divided into two different types: specific pathological internet use (S-PIU) and generalized pathological internet use (G-PIU). S-PIU refers to addiction toward specific functions of the internet, including online sexual material, online gambling, online auctions, online stock trading, etc. On the other hand, G-PIU refers to addiction toward general internet usage, including wasting time online without any specific function. Online chatting is considered to be associated with G-PIU (22). Since this study aims to understand individuals who prefer online social interaction and their intention to accept virtual humans as their interaction targets, it specifically focuses on G-PIU.

Regarding G-PIU, it is considered that psychopathological factors, including depression, social anxiety, and substance dependence, can impact maladaptive cognitions, which may lead to G-PIU (22). This has been consistently supported by studies indicating that social factors such as depression, anxiety, loneliness, and deficiencies in social relationship skills influence maladaptive psychological states and G-PIU (23–27). Given the relationship between psychopathological factors and G-PIU, there are studies explaining that individuals with G-PIU tend to prefer online social interaction and manage their mood through internet use (10, 11).

The relationship between preference for online social interaction and mood management with G-PIU can be explained by the fact that psychopathological factors induce G-PIU. The definition of preference for online social interaction can be elucidated by Caplan (25), who defines it as “beliefs that one is safer, more efficacious, more confident, and more comfortable with online interpersonal interactions and relationships than with traditional face-to-face social activities” (p. 629). With this definition, we can understand that individuals suffering from psychopathological factors perceive these factors to have less of an impact on them when they are online. Therefore, it can be inferred that individuals with psychopathological factors prefer online social interactions relative to offline social interactions.

Regarding mood management, it can be explained that in the online environment where psychopathological factors have less influence, individuals with G-PIU can alleviate stress, control self-presentation, and feel confident, which aids in mood management (10, 26, 28). In this study, mood management is defined as the intentional motivation to use online chatting for the purpose of managing one’s mood (10, 24, 26, 29). With these definitions, it can be assumed that individuals who prefer online social interactions use online platforms for the purpose of mood management. Specifically, this study assumes that individuals who prefer online social interaction will use online chatting for mood management. Regarding online chatting for mood management, this study further divides the types of online chatting that individuals with a preference for online social interaction seek. Specifically, it is assumed that when someone with a preference for online social interaction seeks mood management, they may seek either casual chats with friends or therapeutic chats. Based on this premise, this study presents the following research hypotheses.

*H1-1*: Preference for online social interaction has positive impact on using casual online chatting for mood management purpose.

*H1-2*: Preference for online social interaction has positive impact on using therapeutic online chatting for mood management purpose.

### Technology Acceptance Model

Technology Acceptance Model (TAM) was proposed by Davis (15) and is frequently adopted in research to explain what affects consumers’ intention to use technological products or services (30, 31). Since TAM’s main purpose is to explain technology acceptance, it is applied in various research fields where technology is applied. As the healthcare field actively integrates technology to efficiently operate healthcare environments and enhance consumers’ healthcare experiences, research in healthcare also actively applies TAM to explain technology acceptance intentions. This includes telemedicine technology, internet-based health applications, mobile medical information systems, and personal digital assistants (32–38). Therefore, there have been numerous attempts to verify potential consumers’ acceptance intentions of technology-based personalized digital healthcare services.

Representatively, there are numerous studies examining the application attempts and acceptance intentions of chatbot services, which can be considered similar to the main object of this study: virtual humans who can chat as friends for mood management. In particular, in the healthcare field, chatbots are utilized for mental health counseling (39), health and wellness mobile chatbots (37), and promoting health behaviors (40). However, these chatbots differ from the current study in that they are developed for medical purposes. On the other hand, this study aims to investigate whether users will voluntarily accept virtual humans as friends for mood management to enhance their psychological well-being. This study represents an early exploration of this approach, and thus applies TAM to exploratively verify users’ acceptance intentions of virtual humans as friends for mood management.

The basic framework of the TAM presents perceived usefulness and perceived ease of use as variables that influence attitudes, which are determinants of technology acceptance (41). Perceived usefulness can be defined as the degree to which someone believes that interacting with the technology is beneficial to themselves, and perceived ease of use can be defined as someone’s belief that the technology can be used without any effort (31). Since this study focuses on virtual human technology and aims to examine whether people who have a preference for online social interaction and use online chatting for management will accept virtual humans as their friends to assist with their mood management, perceived usefulness and perceived ease of use from TAM are applied to explain this acceptance relationship.

This study assumes that individuals who prefer online social interaction and use online chatting for mood management might perceive virtual humans as useful and easy to use. People with a preference for online social interaction often experience difficulties in face-to-face interpersonal interactions. As mentioned earlier, according to the definition of preference for online social interaction, individuals who prefer online social interaction believe that they are safer, more efficacious, more confident, and more comfortable with online interpersonal interactions and relationships than with traditional face-to-face social activities (25). This implies that they consider online interactions, which do not require face-to-face interaction, to be safer, more efficacious, confident, and comfortable. Therefore, if the interaction involves a non-human object, they may feel even safer, more efficacious, confident, and comfortable interacting with it. This suggests that if they need someone to chat with to manage their mood, a non-human and communicable object can be perceived as useful to them. Moreover, the presence of virtual humans, potential objects to chat with, appearing in social media in daily life, may make them perceive virtual humans as easy to use.

*H2-1*: Using casual online chatting for mood management purposes has a positive impact on the perceived usefulness of virtual humans as online chatting objects.

*H2-2*: Using casual online chatting for mood management purposes has a positive impact on the perceived ease of use of virtual humans as online chatting objects.

*H3-1*: Using therapeutic online chatting for mood management purposes has a positive impact on the perceived usefulness of virtual humans as online chatting objects.

*H3-2*: Using therapeutic online chatting for mood management purposes has a positive impact on the perceived ease of use of virtual humans as online chatting objects.

Since TAM’s main purpose is to explain the process by which people develop an intention to accept technology, the ultimate goal is to determine whether they have the intention to accept the technology or not. This study also aims to verify whether the intention to accept virtual humans arises based on perceived usefulness and perceived ease of use. Based on TAM, it is assumed that individuals who use casual online chatting for mood management purposes among those who prefer online social interaction might perceive virtual humans as effective and efficient objects with whom they can communicate online when attempting to manage their mood. According to this reasoning, it is expected that they may ultimately have the intention to accept virtual humans as friends who can help with their mood management. Based on this argument, this study presents the following research hypotheses.

*H4*: The perceived usefulness of virtual humans as online chatting objects has a positive impact on the intention to accept virtual humans as friends for their mood management.

*H5*: Perceived ease of use of virtual humans as online chatting objects has a positive impact on the intention to accept virtual humans as friends for their mood management.

### Uncanny Valley Effect

The Uncanny Valley effect, first proposed by Mori (16), describes the phenomenon in which people initially feel an increased affinity for objects that resemble humans or behave like humans, but then experience a sense of unease when the objects become almost indistinguishable from humans. This effect is characterized by a decrease in affinity for objects that closely resemble humans (12). MacDorman and Chattopadhyay (42) argue that if artificial objects designed to be human-like, such as animated characters or android robots, fail to be distinguished from humans, they are inherently inconsistent in realism. MacDorman and Chattopadhyay (42) propose that this inherent inconsistency in realism may lead to feelings of coldness and eeriness, based on the realism inconsistency theory (42). However, technological advancements are bringing non-human creatures closer to a level that is almost indistinguishable from humans.

The key variable that explains the uncanny feeling in the Uncanny Valley Effect (UVE) is perceived eeriness. Perceived eeriness, as described in UVE, refers to the feeling of disgust and strangeness that occurs when the details of a human-like object do not quite conform to expectations (16). UVE elucidates how people perceive a particular human-like object using the variable “perceived eeriness.” One study by Shin, Song, and Chock (13) further verifies that perceived eeriness affects people’s decisions regarding whether to accept a person as a friend on social media. Specifically, according to Shin, Song, and Chock (13), perceived eeriness has an impact on friendship decisions among avatar users in a virtual social networking service. The study results show that perceived eeriness has a negative impact, leading to the rejection of friend requests sent from unknown avatar users.

This study also aims to determine whether social media users may accept virtual humans as their friends for mood management. It is assumed that the perceived eeriness of virtual humans can influence users’ decisions regarding whether to adopt them as friends. Even if users perceive virtual humans in social media as useful and easy to use, if they perceive them as eerie, they may reject accepting them as friends. Based on this assumption, this study aims to determine whether the perceived eeriness of virtual humans moderates the relationship between perceived usefulness and intention to accept, as well as perceived ease of use and intention to accept. The hypotheses are as follows.

*H6*: Perceived eeriness moderates the effect between the perceived usefulness of virtual humans as online chatting objects and the intention to accept virtual humans as friends for their mood management.

*H7*: Perceived eeriness moderates the effect between the perceived ease of use of virtual humans as online chatting objects and the intention to accept virtual humans as friends for their mood management.

**Figure 1:**
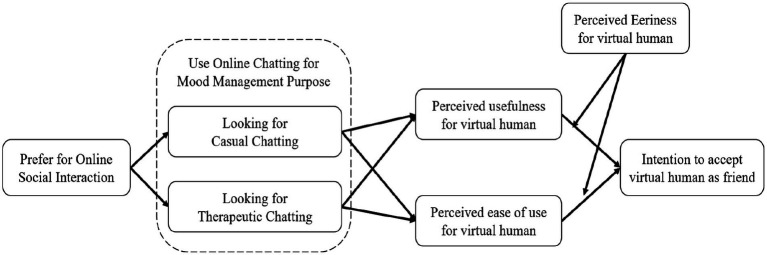
Research model.

Based on the above literature review and hypotheses, this study presents a research model as shown in Figure 1.

## Research method

### Data collection

All experiment procedures were approved by the Sungkyunkwan University’s Institutional Review Board (IRB No. SKKU 2023-02-036). Participants filled out an informed consent form prior to participation. The survey for the study was conducted from February 07 to February 10, 2023. It was commissioned to a professional research institution and conducted online through a web questionnaire.

Since this study aims to understand whether people who prefer online social interaction can accept virtual humans on social media as their friends for mood management, responses were collected from Instagram users in South Korea, where virtual humans that closely resemble humans are actively featured with various concepts. Instagram was selected due to its unique characteristics as visual-first, aesthetics-focused social media platform that emphasizes personal branding, visual storytelling, and community building. The integration of e-commerce features and the spontaneity and authenticity provided by Stories make Instagram an ideal environment to observe user interactions with virtual influencers.

To collect data, participants were instructed to visit the designated Instagram account feed of Rozy, South Korea’s pioneering virtual influencer. Rozy represents a historical milestone as the first virtual influencer in the country, emphasizing lifelike realism, relatability and a diverse representation of beauty. A fashion and lifestyle icon, Rozy engages primarily with Gen Z audiences through targeted marketing partnerships and brand collaborations. Participants were asked to browse the feed for 1 min, clicking on at least 3 posts to immerse themselves in the influencer’s content.

This research method of guiding participants to respond after browsing is a frequently used approach to examine users’ responses after using a specific online service (43, 44). After viewing the virtual human, participants were asked to answer questions. A total of 743 responses were utilized for final data analysis. Summarized demographic characteristics are shown in Table 1.

**Table 1:** Demographic characteristics.

Type		Frequency	Rate (%)
Gender	Male	364	48.9
	Female	379	51.0
Age	14 ~ 19	146	19.6
	20 ~ 29	146	19.6
	30 ~ 39	149	20.0
	40 ~ 49	150	20.1
	50 ~ 59	152	20.4
Education	Less than high school graduate	104	14.0
	High school graduate	83	11.2
	Currently enrolled in college	69	9.3
	College graduate	411	55.3
	Currently enrolled in graduate school	14	1.9
	Graduate school graduate	62	8.3

### Measurements

For the measurement, questionnaires for each variable are shown in Table 2. Prefer for online social interaction (3 questionnaires), online chatting mood regulation (including ‘looking for casual chatting’; 3 questionnaires and ‘looking for therapeutic chatting’; 3 questionnaires) measurements were based on research by Caplan (10). Perceived usefulness (3 questionnaires) and perceived ease of use (4 questionnaires) variables from TAM’s measurements were based on Fernandes and Oliveira (31). Perceived eeriness (3 questionnaires) measurements were based on (47). Lastly, the intention to accept virtual characters as a friend (3 questionnaires) measurements were based on Fernandes and Oliveira (31).

**Table 2:** Measurements.

Variable		Questionnaire	References
Prefer for Online Social Interaction		I prefer online social interaction over face-to-face communication. Online social interaction is more comfortable for me than face-to-face interaction. I prefer communicating with people online rather than face-to-face.	(10)
Online Chatting Mood Regulation	Looking for casual chatting	I have used online chatting to casually talk with others when I was feeling isolated. I have used casual online chatting to make myself feel better when I was down. I have used casual online chatting to make myself feel better when I’ve felt upset.	
	Looking for therapeutic chatting	I have used online chatting for therapeutic talk when I was feeling isolated. I have used therapeutic online chatting to make myself feel better when I was down. I have used therapeutic online chatting to make myself feel better when I’ve felt upset.	
Perceived Usefulness		I think the interaction with virtual human is useful for my mood management. I think chatting with a virtual human can help me with my mood management. I think the virtual human is useful to chat with for my mood management.	(31)
Perceived Ease of Use		I find chatting with a virtual human easy. I think I can chat with the virtual human without any help. I think I can chat with the virtual human without any effort. I think I can easily interact with the virtual human.	
Perceived Eeriness		Uninspiring ↔ Spine-tingling Bland ↔ Uncanny Unemotional ↔ Hair-raising	(47)
Intention to accept virtual human as a friend		I plan to accept the virtual human as a friend for my mood management in the future. I will try to accept the virtual human as a friend for my mood management in the future. I intend to accept the virtual human as a friend that can help with my mood management in the future.	(31)

## Results

### Measurement model evaluation

Measurement model evaluation is based on reliability and validity evaluation after confirming factor analysis of the measurement model.

#### Reliability and validity evaluation

The reliability was evaluated using the Cronbach’s Alpha coefficient and the Internal Composite Reliability. The Cronbach’s Alpha values for all latent variables exceed the baseline of 0.70, and the composite reliability values also exceed the baseline of 0.70. Therefore, the metrics are evaluated to have internal consistency reliability. The values of Cronbach’s Alpha and composite reliability are summarized in Table 3.

**Table 3:** Cronbach alpha and internal composite reliability values.

	Prefer for online social interaction	Online chatting mood regulation		Perceived usefulness	Perceived ease of use	Perceived eeriness	Intention to accept
		Casual chatting	Therapeutic chatting				
Cronbach α	0.879	0.928	0.945	0.938	0.911	0.710	0.944
Omega	0.879	0.930	0.945	0.939	0.912	0.721	0.945
Omega 1	0.879	0.930	0.945	0.939	0.912	0.721	0.945
Omega 2	0.879	0.931	0.946	0.940	0.912	0.722	0.945

The indicator reliability of measurements was verified by standardized estimates through the calculation of standardized coefficients. For standardized estimates, it was found that all of the coefficient values exceeded the baseline of 0.708, except for Perceived Eeriness measurements; PE 1 (0.626) & PE 2 (0.626). However, in previous studies, standardized coefficient values up to 0.60 are acceptable (45), so this study retained PE 1 and PE 2 without removal. The indicator reliability figures are summarized in Table 4.

**Table 4:** Indicator reliability.

Variable		Measurement	Est. std	*z*	*p*
Preference for Online Social Interaction		POSI 1	0.865	51.345	0.000
		POSI 2	0.805	38.055	0.000
		POSI 3	0.857	40.101	0.000
Online Chatting Mood Regulation	Looking for casual chatting	CC 1	0.891	69.103	0.000
		CC 2	0.949	89.095	0.000
		CC 3	0.870	60.004	0.000
	Looking for therapeutic chatting	TC 1	0.908	70.431	0.000
		TC 2	0.954	91.469	0.000
		TC 3	0.910	68.179	0.000
Perceived Usefulness		PU 1	0.933	119.731	0.000
		PU 2	0.916	94.955	0.000
		PU 3	0.897	76.090	0.000
Perceived ease of use		PEOU 1	0.829	44.298	0.000
		PEOU 2	0.902	71.113	0.000
		PEOU 3	0.904	69.940	0.000
		PEOU 4	0.765	36.652	0.000
Perceived eeriness		PE 1	0.626	13.604	0.000
		PE 2	0.626	13.551	0.000
		PE 3	0.776	15.520	0.000
Intention to accept virtual human as a friend		ACC 1	0.918	91.670	0.000
		ACC 2	0.923	100.154	0.000
		ACC3	0.928	108.815	0.000

**Figure 2:**
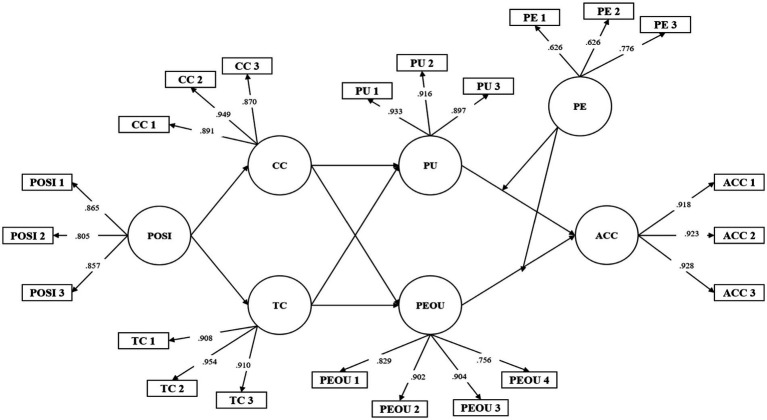
Factor loading diagram.

The significance level of the measurements can also be verified based on the figures provided in the indicator reliability evaluation shown in Table 4 and visualized in Figure 2. In the indicator reliability evaluation, when the z-value exceeds the baseline of 2.0 and the *p*-value is 0.000, convergent validity is achieved.

In covariance-based structural equation modeling, the average variance extracted square root of the latent variable must be higher than the correlation coefficient of other latent variables. The analysis reveals that the correlation coefficients between latent variables were found to be smaller than the square root of the average variance extraction, thereby confirming discriminant validity. Table 5 presents the matrix between the square root of the average variance extraction of latent variables and the correlation coefficient between latent variables.

**Table 5:** Matrix between square root of AVE and correlation coefficient.

	POSI	CC	TC	PU	PEOU	PE	ACC
POSI	**0.843** ^*^						
CC	0.338	**0.904** ^*^					
TC	0.349	0.602	**0.924** ^*^				
PU	0.301	0.360	0.385	**0.916** ^*^			
PEOU	0.148	0.217	0.147	0.494	**0.852** ^*^		
PE	−0.014	0.058	0.136	−0.009	−0.083	**0.679** ^*^	
ACC	0.258	0.322	0.376	0.792	0.463	−0.044	**0.923** ^*^

* Square root of AVE.

#### Structural model evaluation

Model fit was evaluated using the ‘Lavaan’ package, and the validity of the model has been verified. The GFI (Goodness-of-fit index) is 0.897, which falls short of the baseline (≥0.9). However, other key indices indicate a good fit: the CFI (Comparative Fit Index) is 0.944, the TLI (Turker-Lewis Index) is 0.935, and the NFI (Normed Fit Index) is 0.930, all of which exceed the baseline (≥0.9). Additionally, the RMSEA (Root Mean Square Error of Approximation) is 0.071, which is below the baseline (≤0.08). The model fit indices and their respective figures are summarized in Table 6.

**Table 6:** Model fit.

	CFI	TLI	GFI	NFI	RMSEA
Criteria	≥0.9	≥0.9	≥0.9	≥0.9	≤0.08
Research model	0.944	0.935	0.897	0.930	0.071

### Hypotheses verification

**Figure 3:**
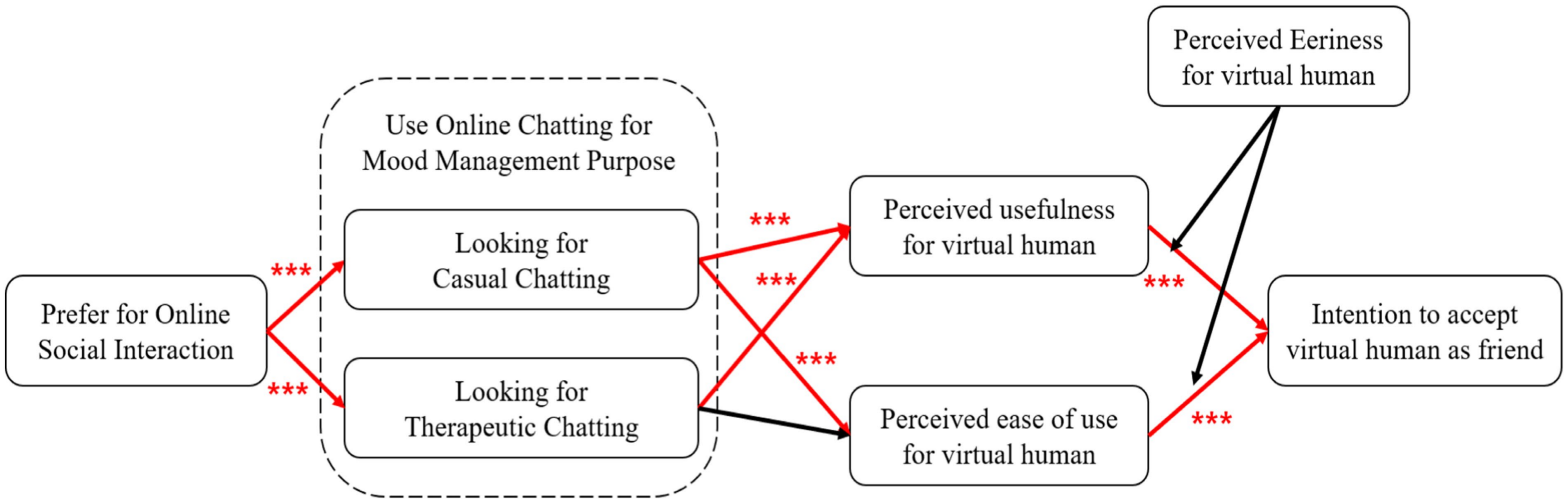
Hypotheses verification result.

The hypotheses were verified by path coefficient and significance. The results of hypothesis verification are summarized in Table 7 and Figure 3 with path coefficients and significance levels.

**Table 7:** Hypotheses verification result.

Hypotheses		Path	Est. std	*z*	*p*	Result	
H1	1	POSI → CC	0.368	10.423	0.000***	Supported	Supported
	2	POSI → TC	0.377	10.829	0.000***	Supported	
H2	1	CC → PU	0.217	6.042	0.000***	Supported	Supported
	2	CC → PEOU	0.200	5.255	0.000***	Supported	
H3	1	TC → PU	0.282	8.013	0.000***	Supported	Partially supported
	2	TC → PEOU	0.039	1.001	0.317	Rejected	
H4		PU → ACC	0.757	42.087	0.000***	Supported	Supported
H5		PEOU → ACC	0.124	4.583	0.000***	Supported	

PE moderating effect						
PE moderating effect		Coefficient	*z*	*p*	Result	
H6	PE → PU–ACC	0.040	1.692	0.091	Rejected	Rejected
H7	PE → PEOU–ACC	−0.033	−0.885	0.376	Rejected	

Hypothesis 1, which aimed to examine whether people who prefer online social interaction seek online chatting for mood management purposes, was supported. Specifically, Hypothesis 1–1, which suggested that people who prefer online social interaction seek casual online chatting for mood management purposes, was supported (*p* = 0.000***). Similarly, Hypothesis 1–2, indicating that people who prefer online social interaction seek therapeutic online chatting for mood management purposes, was also supported (*p* = 0.000***).

Hypotheses 2 and 3 were formulated to investigate whether individuals seeking mood management through casual or therapeutic online chatting perceive virtual humans as useful and easy to use. Specifically, Hypothesis 2 aimed to determine whether those engaging in casual online chatting for mood management perceive the usefulness (H2-1) and ease of use (H2-2) of virtual humans as online chatting objects. Both H2-1 (*p* = 0.000***) and H2-2 (*p* = 0.000***) were supported. However, Hypothesis 3, which aimed to explore whether individuals engaging in therapeutic online chatting for mood management perceive the usefulness (H3-1) and ease of use (H3-2) of virtual humans as online chatting objects, yielded mixed results. While H3-1 (*p* = 0.000***) was supported, H3-2 (*p* = 0.317) was rejected.

Hypotheses 4 and 5 aimed to investigate the impact of perceived usefulness and perceived ease of use on the intention to accept virtual humans as friends for mood management. Both H4 (*p* = 0.000***) and H5 (*p* = 0.000***) were supported.

In addition, this study tested hypotheses 6 and 7 to determine if there is a moderating effect of perceived eeriness between perceived usefulness and intention to accept (H6) and between perceived ease of use and intention to accept (H7). Moderating effect analysis examines whether the interaction effect between the independent variable (X) and the moderating variable (Z) on the dependent variable (Y) is statistically significant. The moderating effect was assessed using the ‘Lavaan’ package provided by R Studio. The results indicate that there is no moderating effect. Therefore, both H6 (*p* = 0.091) and H7 (*p* = 0.376) were rejected.

## Discussion and conclusion

This paper focuses on the advancement of technology that has opened up possibilities for its application in various fields, particularly in healthcare. Notably, there have been attempts to utilize virtual humans as virtual healthcare workers in healthcare settings. In this context, the study aims to explore the potential application of virtual humans, which closely resemble humans and are often seen in social media, in assisting individuals with managing their mood for psychological well-being in daily life. As an early-stage exploration of the idea of using virtual humans for mood management, the study establishes and verifies a research model based on the Problem Behavior Theory, Technology Acceptance Model, and Uncanny Valley Effect. The discussion section of this study will explore the implications of the rejected hypotheses.

First, Hypothesis 3–2 was rejected. Hypothesis 3 aimed to investigate whether individuals preferring online social interaction would seek therapeutic online chatting for mood management and, if so, whether they would perceive virtual humans as useful and easy to use for online chatting. Specifically, Hypothesis 3–2, which suggested that those seeking therapeutic online chatting would find virtual humans easy to use as online chatting objects, was rejected. While Hypothesis 3–1, indicating that individuals would perceive virtual humans as useful for therapeutic purposes, was supported, it suggests that people perceive virtual humans could work therapeutically if they intend to, but they may not know how to utilize them in that manner. This might be attributed to the current role of virtual humans on social media, primarily as influencers, making it challenging for users to envision them as therapeutic online chatting objects. However, with ongoing research on the ethical implications of therapists’ activities on social media (46), the emergence of social media virtual influencers with therapeutic purposes could potentially increase ease of use.

Secondly, the moderating effect of perceived eeriness of virtual humans between perceived usefulness and intention to accept (H6) and perceived ease of use and intention to accept (H7) was both rejected. Perceived eeriness is a variable derived from the uncanny valley effect, indicating people’s discomfort toward entities that resemble humans to a certain degree. Previous studies have shown that when people experience eeriness or discomfort toward someone, they tend to hesitate to form friendships in an online environment (13). However, the results of this study differ from previous research findings, suggesting the need for further investigation. According to the uncanny valley effect, if a human-like entity surpasses a certain threshold known as the “uncanny valley,” people cease to feel eeriness or discomfort. However, since this study focused on the use of virtual humans for mood management purposes, it remains unclear whether the uncanny valley effect does not apply even when used in other domains. This underscores the necessity for future research.

Academically, this study offers a unique perspective on existing theory. Specifically, it reframes problematic behavior, as defined by the Problematic Behavior Theory, not merely as negative but as potentially beneficial in terms of technological utilization. For instance, the preference for online social interaction over offline, in-person interaction is traditionally viewed as problematic. However, this study posits that individuals who favor online social media may be more adept with technology and may effectively manage their mood through online chatting. By applying this alternative viewpoint, the study contributes to the academic discourse, enriching the validity of the theory and paving the way for further research in diverse contexts of media technology usage.

Secondly, this study suggests the possibility that recent advancements in virtual human development technology may have mitigated the uncanny valley effect. Initially, based on the uncanny valley effect hypothesis, this study posited that if individuals perceive eeriness toward virtual humans, it might hinder acceptance. However, the findings of this study suggest that advancements in technology may have enabled human-like creatures to transcend the uncanny valley zone. This hypothesis warrants further investigation in future studies.

Lastly, this study expands the research on virtual humans from a focus on their commercial or marketing roles to exploring their potential social and emotional roles. This suggests a new direction and topic for future research, particularly emphasizing the need for studies that consider various aspects, such as the platforms where virtual humans appear, their physical characteristics, and the traits of users. This paper provides foundational material for future research.

Practically, first, this paper offers implications by suggesting how virtual humans can play a role in having a social impact. In this study, virtual humans on social media, originally designed for marketing and advertising purposes to generate profit from a business perspective, are evaluated as having the potential to help social media users manage their mood. While further research is needed, this study presents the possibility of social impact beyond mere commercial use and the potential for business expansion.

Secondly, the findings demonstrate that potential users are willing to accept virtual humans as friends on social media for mood management. Particularly noteworthy is the result that people expect casual online chatting with virtual humans for mood management. This suggests that virtual humans could become a practical business model in the future.

Finally, this study proposes a practical business design for future virtual humans. For example, if users request a chat tailored to their mood, it would be possible to add mental care services through casual chatting. Such services could create new opportunities in the fields of mental health management or personalized customer service.

This paper has several limitations. First, the data analysis was based solely on Instagram users. Second, the virtual human ‘Rozy’ was selected to help participants imagine whether they would accept her as a friend for mood management. Third, the study did not distinguish between participants who are currently taking steps to improve their mental health and those who are simply interested in mood management. By collecting responses from a single social platform and a single virtual human without considering the participants’ characteristics, the scope and generalizability of the findings were limited. However, as this study serves as an initial exploration of the topic, its value lies in investigating the potential of virtual humans in social media. Future research should address these limitations to provide more generalized insights. Additionally, future studies should consider understanding the basic messaging formats of social media platforms and their sub-features. Moreover, it will be important to categorize the visual, auditory, and background factors of virtual influencers and gather responses based on these distinctions. Finally, a more detailed analysis is needed to differentiate participants based on whether they are actively using methods to improve their mental health or are simply interested in improving their mood management, allowing for a more focused understanding of their reactions.

## Data Availability

The dataset presented in this study can be found in online repositories at: https://osf.io/jq8nc/.
